# Overcoming Barriers for siRNA Therapeutics: From Bench to Bedside

**DOI:** 10.3390/ph13100294

**Published:** 2020-10-07

**Authors:** Muhammad Imran Sajid, Muhammad Moazzam, Shun Kato, Kayley Yeseom Cho, Rakesh Kumar Tiwari

**Affiliations:** 1 Center for Targeted Drug Delivery, Department of Biomedical and Pharmaceutical Sciences, Chapman University School of Pharmacy, Harry and Diane Rinker Health Science Campus, Irvine, CA 92618, USA; sajid@chapman.edu (M.I.S.); skato@chapman.edu (S.K.); yecho@chapman.edu (K.Y.C.); 2 Faculty of Pharmacy, University of Central Punjab, Lahore 54000, Pakistan; moazzamshakeel91@gmail.com

**Keywords:** siRNA delivery systems, barriers to siRNA delivery, endosomal escape, reticuloendothelial system entrapment, renal clearance, off-target effects, immune system activation, chemical modifications, membrane impermeability

## Abstract

The RNA interference (RNAi) pathway possesses immense potential in silencing any gene in human cells. Small interfering RNA (siRNA) can efficiently trigger RNAi silencing of specific genes. FDA Approval of siRNA therapeutics in recent years garnered a new hope in siRNA therapeutics. However, their therapeutic use is limited by several challenges. siRNAs, being negatively charged, are membrane-impermeable and highly unstable in the systemic circulation. In this review, we have comprehensively discussed the extracellular barriers, including enzymatic degradation of siRNAs by serum endonucleases and RNAases, rapid renal clearance, membrane impermeability, and activation of the immune system. Besides, we have thoroughly described the intracellular barriers such as endosomal trap and off-target effects of siRNAs. Moreover, we have reported most of the strategies and techniques in overcoming these barriers, followed by critical comments in translating these molecules from bench to bedside.

## 1. Introduction

**Figure 1:**
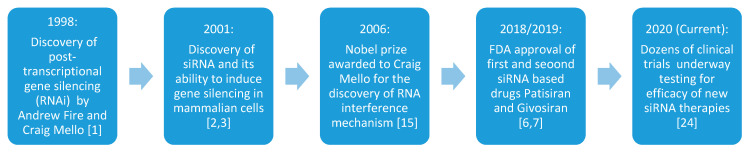
Timeline highlighting the major historical events leading up to siRNA therapeutics.

In 1998, Andrew Fire and Craig Mello published a seminal paper in which they discovered the phenomenon of post-transcriptional gene silencing (PTGS) and termed it as RNA interference (RNAi) [1]. Later, in 2001, two research groups reported that 21 to 22 nucleotide(nt) double-stranded RNAs, popularly known as small interfering RNAs (siRNAs), can induce gene silencing in mammalian cells without causing nonspecific interferon response [2,3]. siRNAs, composed of a duplex of 20 to 24 nucleotide base pairs, work by interacting with the RNAi pathway enzyme dicer for cleavage. The single-stranded antisense strand complementary to the target mRNA is handed to the RNA- induced silencing complex (RISC) loading complex (RLC), which destroys the target mRNA, hence silencing the target gene or, in other words, inhibit the formation of the target protein [4]. Section 2 further explains the mechanism of siRNA in detail. These siRNAs later kicked off a revolution in biology due to their potential to inhibit virtually all genes by a base sequence of target mRNA alone, which gives RNAi several advantages over small-molecule drugs as a therapeutic strategy [5]. Approval of the first siRNA-based drug patisiran (Onpattro™) by the U.S. Food and Drug Administration (FDA) in 2018 for polyneuropathy of hereditary transthyretin-mediated amyloidosis has fostered a new interest by the pharmaceutical as well as academic research groups in RNAi based drugs [6]. Recently in 2019, givosiran (Givlaari™) has been approved by the US FDA for acute hepatic porphyria [7], which has further reinforced the confidence in siRNA based products. A timeline of these events is shown below in Figure 1.

Furthermore, dozens of siRNA-based products are already in different stages of clinical trials [8]. For instance, an investigational RNAi product targeting glycolate oxidase, termed lumasiran, is currently in phase 3 clinical trial for the treatment of primary hyperoxaluria type 1 (PH1), in which calcium oxalate crystals are formed in the kidney and urinary tract, leading to recurrent kidney stones and nephrocalcinosis [9]. Lumisiran has shown promising results with encouraging safety and tolerability profile with no serious adverse event during the study time [10].

Similarly, fitusiran, another RNAi therapy, targets antithrombin (AT) in the liver, silencing AT gene expression, and prevents AT synthesis. Fitusiran is undergoing phase III clinical trial as a prophylactic treatment of hemophilia [11,12]. Also, vutrisiran is undergoing a phase-III clinical trial for the therapy of transthyretin amyloidosis (ATTR) with cardiomyopathy [13]. See Section 4 for more details on current clinical trials on siRNA therapeutics. Although several studies have shown the significant potential of siRNA therapeutics in-vitro, yet systemic siRNA therapy faces several extracellular as well as intracellular barriers for translation of siRNA therapeutics from bench to bedside. Several reviews have been published to address this topic comprehensively, particularly on the applications of siRNA therapeutics [14,15,16]. However, there is an urgent need for a thorough investigation of barriers reported in the scientific literature. Also, there have been several studies that describe the methods and techniques in overcoming these barriers for the translation of siRNA therapeutics to the clinics. Briefly, the major extracellular barriers are enzymatic degradation of siRNAs by serum endonucleases and RNAases, rapid renal clearance of siRNA delivery system, impermeability of siRNA to the biological membranes, activation of the immune system, plasma protein sequestration, and capillary endothelium crossing. In contrast, the intracellular obstacles to siRNA action are the endosomal trap, arrival at the correct intracellular site of action (cytosol), and off-target effects. In this review, we attempted to describe comprehensively these barriers and the milestones achieved in addressing the obstacles followed by critical commentary on innovation and prospects.

## 2. Mechanism of Action of siRNA Induced RNA Interference (RNAi)

**Figure 2:**
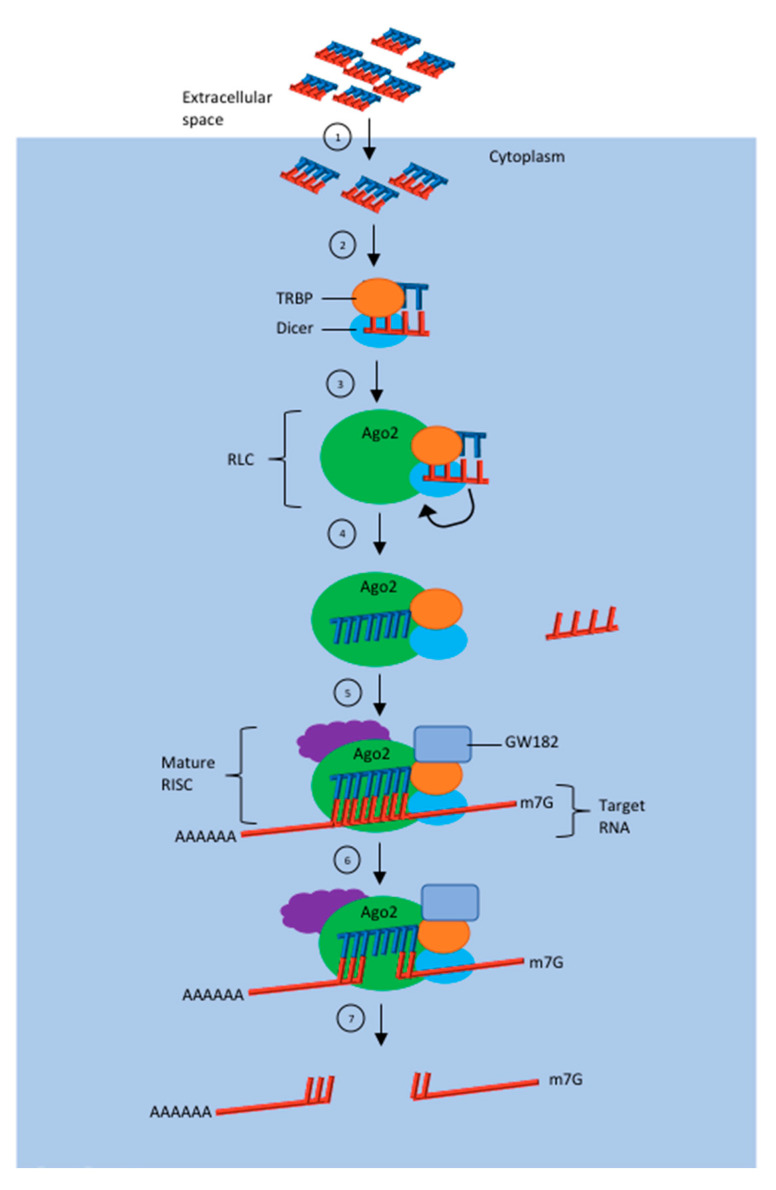
General mechanism of action for siRNA induced gene silencing. (1) Synthetic siRNA successfully enters the cell via endocytosis. (2) siRNAs will then travel in the cytosol until it comes into contact with cytosolic RNAi enzymes–dicer and Tar RNA Binding Protein (TRBP) to form RISC Loading Complex (RLC). (3) Strand selection is done. (4) Production of mature RISC following successful guiding strand selection. (5) siRNA guiding strand with full complementarity to a single target mRNA induces potent and targeted gene silencing. (6) Mature RISC can regulate gene expression via inhibition of mRNA translation, inducing mRNA sequestration in cytoplasmic P bodies and or GW bodies to promote mRNA degradation. (7) Degraded mRNA is no longer useful and cannot go through translation to produce protein.

siRNA therapies take advantage of a naturally occurring phenomenon known as RNAi. The mechanism of action begins with the synthesis of a generally perfectly base-paired dsRNA ranging from 15 to 30 bp in length [15]. The length of dsRNA plays a crucial role as siRNA smaller than 15 bp does not incorporate itself with the RNAi machinery. Additionally, siRNA larger than 30 bp can lead to cytotoxicity and nonspecific interactions via the protein kinase R (PKR) pathway [17]. Following the successful entrance into the cytosol via endocytosis, synthetically produced siRNAs can interact directly with cytosolic RNAi enzymes known as Dicer and Tar RNA Binding Protein (TRBP) [18]. siRNA larger than 21 bp will interact with the Dicer enzyme that cleaves and hands it off to the RNA-induced silencing complex (RISC) loading complex (RLC) [4,19]. In comparison, siRNA shorter than 21 bp can bypass Dicer cleavage, and through interactions mediated by TAR RNA-binding protein, it can successfully enter RISC. Prior to mature RISC production, the complex must successfully select the guide strand between the antisense and sense strand. The antisense strand is the guide strand of choice, and through chemical modifications, biases towards its selection are possible. The siRNA guide strands, or antisense strands usually have exact 100% complementarity to a single target mRNA which allows for the potent and successful silencing of the gene. Following the successful loading of the guiding stand, TRBP and Dicer have the ability to dissociate from RISC. Ago2, which is an enzyme that joins and associates itself with the RLC, plays an important role for gene silencing as it has intrinsic silencer activity to efficiently cleave the mRNA targets [20]. Gene expression is regulated through the inhibition of mRNA translation in the form of inducing mRNA sequestration in cytoplasmic processing bodies (P bodies) and/or GW bodies, which ultimately promotes mRNA degradation and directing transcriptional gene silencing of the target gene [21,22,23]. Figure 2 illustrates the mechanism of siRNA action.

## 3. Advantages of siRNA over Other RNAi Therapeutics

There are several types of RNAi therapeutics: micro RNA (miRNA), short hairpin (shRNA), and siRNA. miRNA is a single-stranded RNA that has the stem-loop structure and binds imperfectly to mRNA. Due to this characteristic, miRNA can degrade many sets of similar mRNAs, causing toxicity [24]. shRNA has a tight hairpin structure, making it more complicated than other RNAi therapeutics. It requires the promoter to be expressed and located in the nucleus to act appropriately [25]. Lastly, siRNA is the short stretch double-stranded RNA that can degrade the complementary mRNA. siRNA has high transfection efficacy and fewer obstacles, making the best fit for RNAi therapeutics [26]. Table 1 illustrates the different types of RNAi therapeutics.

## 4. siRNA-Based Therapeutics in Clinical Trials

Many siRNA-based therapeutics are making their way onto the clinical trial stage for a multitude of diseases. The following clinical trials are specific to therapeutics to treat cancer. Although many of these therapeutics did not make their way to Phase II trials, some have been more recently modified, making its way back to the clinical stage. An investigational siRNA therapeutic is CALAA-01 from Calando Pharmaceuticals [18]. This therapeutic is a four-component polymer-based nanoparticle siRNA delivery system around 75 nm in diameter [27,28]. The complex consists of a linear, cationic cyclodextrin-based polymer known as adamantane polyethylene glycol or PEG, which is incorporated to provide steric stabilization, along with a human transferrin protein-targeting agent and a siRNA that targets the reduction in the M2 subunit of ribonucleotide reductase (RRM2) expression [18]. Existing data reveals that RRM2 plays a large role during nucleic acid metabolism and is upregulated in many tumor types [18,27,28,29]. It is proposed that the suppression of RRM2 will lead to cell cycle arrest and cell death [30].

Another therapeutic investigation was from Alnylam Pharmaceuticals named ALN-VSP. This siRNA-based therapeutic is a nearly neutral nanoparticle formulation about 80-100 nm in diameter, with two different chemically modified siRNAs targeting different proteins, in a 1:1 molar ratio [31,32]. The siRNAs individually target vascular endothelial growth factor A (VEGFA) and kinesin spindle protein (KSP). Both KSP and VEGFA are highly expressed in a variety of tumor types [31], where VEGF is proposed to modulate tumor angiogenesis or the growth of blood vessels to supply nutrients to tumors, and KSP is essential for mitotic spindle formation in proliferative cells [31]. Targeting of VEGF is proposed to decrease blood supply to the tumor, and KSP is targeted to stop mitotic spindle formation [31].

Atu027, developed by Silence Therapeutics, is a cationic lipoplex-based siRNA delivery system made up of a blunt-ended, chemically modified 23-mer RNA oligonucleotide along with three cationic lipids. The siRNA is created to target protein kinase N3 (PKN3). It is proposed that PKN3 suppression stabilizes vessel integrity and attenuate inflammatory responses in the vasculature of tumors and secondary organ sites via modulation of actin and adherin junction dynamics [18,33,34]. Furthermore, this will ultimately lead to inhibition of mobilization and engraftment of metastatic tumor cells due to the reduction of both tumor and host vascular permeability [18].

Other therapeutics termed TKM-PLK1 from Tekmira carried a siRNA targeting polo-like kinase 1 (PLK1). It is a lipid nanoparticle about 80 nm in diameter targeting PLK1. It is thought to be a key protein during multiple steps in cell division, DNA stability, and DNA repair. It has also been found to be upregulated in many human tumors. It is proposed that the silencing of PKL1 may induce apoptosis and tumor cell death [35].

Another reported therapeutic is PN2258 from ProNAi Therapeutics, which is a liposomal formulation around 130 nm in diameter. It comprises a 24-base chemically unmodified DNA oligonucleotide and four lipid molecules, making the therapeutic unique as it does not contain siRNA [36]. Since it does not contain siRNA, the RNAi mechanism at the RISC complex is not induced. It was included with the other clinical trials because it is systematically administered and is a nucleic acid formulation for anticancer treatment. The nucleic acid portion, also known as PNT100, follows a mechanism where it binds to the 5′-untranscribed regulatory regions of the BCL2 protein gene and blocks transcription via DNA interference rather than RNA interference.

## 5. Barriers to siRNA Delivery

As described earlier, small double-stranded RNA (ds RNA) molecules can efficiently trigger the post-transcriptional gene silencing; still, their delivery has been the major challenge to applying siRNA-based therapeutics in humans [37,38]. Barriers depend on the target organ and the desired route of administration of siRNA. The affluence of siRNA delivery depends on the accessibility of target tissue or organ inside the body. Broadly two administration routes, localized administration, and systemic administration, are used. Localized siRNA delivery has fewer barriers than systemic delivery. In localized delivery of siRNA, therapy is applied directly to the target organ or tissues, offers high bioavailability at the target site, and avoids the barriers that are shown by the systemic administration [39]. Several organs are compliant to local or topical siRNA delivery, including eyes, mucous membranes, localized tumors, and skin [40,41]. Lungs infections can be treated with local siRNA delivery; direct installation of siRNA through intranasal or intra-tracheal routes permits direct contact with epithelial cells of the lungs [42]. Readers are encouraged to read excellent reviews about physical and immunologic barriers of local delivery of siRNA [43,44].

In contrast, the siRNA systemic delivery has various challenges that limit the siRNA bioavailability at the target site [45]. Upon intravenous injection, unmodified naked siRNA is degraded by endogenous enzymes. Also, due to their small size (MW 13 kDa), siRNA is eliminated through kidneys. The siRNA is a negatively charged molecule, and it is almost impossible for siRNA to cross the biological membrane. The half-life of most naked or unmodified siRNA is between 5–10 min [46]. Table 2 illustrates the barriers faced by siRNA to reach its site of action. These barriers limit the application of siRNA-based therapeutics in humans and warrant specific chemical modifications of either siRNA or the delivery system for their widespread use in clinical conditions.

## 6. Intravascular Degradation and Renal Clearance

The first biological barrier after injection is intravascular degradation of siRNA by nucleases enzyme in plasma. The naked or unmodified siRNA is unstable in the systemic circulation and more susceptible to A-type nucleases, which are ubiquitous in intracellular and extracellular space [47]. Besides, fast renal clearance results in the very short half-life of siRNA, ranging from 5–10 min [48,49,50]. Nucleases in plasma or tissues degrade the unmodified siRNA in few minutes to 1 h, potentially limiting the use of siRNA-based therapeutics [51,52]. The reason for these challenges is attributed to their small size, approximately 7 nm in length and smaller molecular weight, i.e., approximately 13 kDa [53]. To this end, siRNA modification is necessary to resist nuclease mediated degradation, reducing siRNA susceptibility towards nucleases and improving their in-vivo properties. However, siRNA modification alone may not be enough to achieve the therapeutic activity. In this way, physical encapsulation of siRNA and chemical modification promotes proper therapeutic activity [54,55].

**Figure 3:**
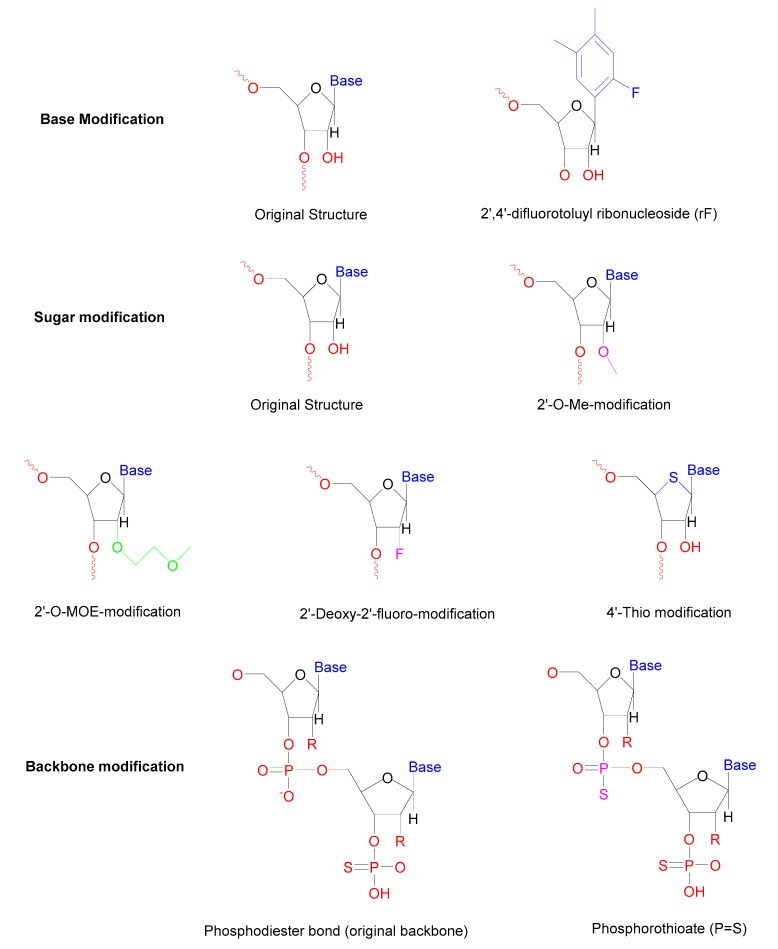
Chemical modifications of siRNA.

Researchers have turned on modifying the sugars, backbone, or bases of oligoribonucleotides for stabilization to overcome intravascular degradation [56]. The modification of base with internal uridine to rF (2,4-difluorotoluyl ribonucleoside) substitutions shows better resistance towards intravascular degradation by nucleases in the serum [57]. Modification of both the sense and antisense strand enhances the nuclease resistance in the serum [58]. In the case of sugar modifications, 4′-thioribonucleosides modification shows higher resistance towards serum nucleases [59]. If ribose 2′-OH group modified with a CH3 group or fluoride atom within both the sense as well as antisense strand, enhanced their resistance towards endonucleases and also improved therapeutic potency of siRNA [60]. A study demonstrated that the siRNA modified with the 2′fluoro group significantly enhanced resistance towards nucleases in plasma but unable to generate prolonged silencing in mice and cell cultures [61]. Figure 3 illustrates these modifications. It has been suggested that modification of siRNA with small molecules such as 2,4-dinitrophenol (DNP) not only increases their ability to resist nucleases, but it also enhances their membrane impermeability [62]. Some reputed modifications of siRNA are presented in Table 3.

Extensive chemical modification of siRNA showed high in-vivo efficacy. For instance, siRNA modification of the sense strand comprised of 2’F-pyrimidines, 2’-deoxypurines with 5’- and 3’-terminal inverted a basic end caps. While, the antisense strand comprised of 2’-fluoro-substituted pyrimidines, 2’-methoxy purines with a single phosphorothioate (PS) linkage at the 3’-terminus, gives prolonged half-life in the serum, reaching 2 to 3 days as compared to naked unmodified siRNA with half-life minutes to less than 1 h [67]. However, the extended chemical modification screening process elaborated that improved serum stability can be achieved by thermodynamic stabilization selectively of some positions within the duplex compared to the thorough substitutions of siRNA with several analogs [68,69].

One of the main mechanisms of removing the siRNA therapeutics from the bloodstream is through urine by glomerular filtration in the kidneys. The pore size of the glomerular filtration barrier is about 8 nm [70], so if the nanoparticles are designed to have a particle size of about 20 nm, this barrier challenge can be addressed efficiently [70]. There are variable reports about the half-life of unmodified siRNA in serum, several studies suggest that chemically modified siRNA lasts longer in nuclease rich environment than the unmodified siRNA [71]. Furthermore, increasing its molecular weight by attachment of ligands, incorporating larger particles, or binding to plasma proteins efficiently saves siRNA from elimination [72].

In this context, researchers at Alnylam Pharmaceutical Inc. conjugated the cholesterol with the 3’ end of the siRNA sense strand by a short trans-4-hydroxylprolinol linker. This cholesterol-siRNA conjugation caused the avid binding of modified siRNA with plasma proteins and increased the plasma retention time with the half-life of 90 min [73]. Cholesterol-siRNA conjugation also provided higher resistance to nucleases with greater serum stability and prolonged circulation time [72]. In another study, it observed that cholesterol-siRNA conjugate resulted in greater stability and silenced the endogenous gene expression of apo B protein, which regulates cholesterol metabolism [74]. On the other hand, naked siRNA without cholesterol conjugation did not show any silencing activity inside the cell [74].

Conjugation with polymer, especially PEG, is a common concept to increase the half-life of siRNA in blood and enhance the pharmacokinetic profiles [46,75]. A study demonstrated that conjugating siRNA with 20k PEG resulted in a slower rate of renal clearance (50% remained 1 h after injection) as compared to unmodified siRNA (<10% remained 15 min after injection) [76]. Similarly, the conjugation of PEG at 2-nucleotide 3’-overhang significantly increased the downregulation of PSMA expressing tumors [77].

Besides, siRNA-cationic polymer showed 100-fold more retention time in blood than naked siRNA [78]. The cationic RNAi nanoparticles (NPs) were also shown to be excreted through kidneys. The primary reason may be their interaction with anionic proteins on the capillary wall of the glomerulus; the resulting neutralization may have resulted in the ready release of siRNA on the capillary bed of glomerulus with subsequent excretion. To overcome this barrier, cationic RNAi NPs may also be PEGylated to reduce renal clearance and increase the circulation time of siRNA [55].

Another study showed that cationic comb-type copolymers (CCC), having a considerably higher density of hydrophilic graft chains, increased the stability of siRNA against nuclease and plasma components. The siRNA complexed with CCC acquired considerably longer blood circulation than naked siRNA or complexed with PEI (jetPEI™) [78].

## 7. Activation of the Innate Immune System

The function of innate immunity is to identify the pathogens, eradicate them, and contribute to adaptive immunity. Host cells depend on the pattern recognition receptors that detect infectious and non-self-agents, including lipopolysaccharide, viral RNAs, bacterial DNA, viral glycoproteins, and peptidoglycan [79].

Previously, it was thought that siRNA shorter than 30 nucleotides were small enough to evade the immune system and avoid nonspecific stimulation of interferon response. However, subsequent experiments with short synthetic siRNAs showed that siRNA could activate the immune response and trigger the production of cytokines in-vivo and in-vitro [80]. This innate immune response can be triggered by the siRNA or by the vehicles, including cationic lipids used to deliver siRNA in-vivo [81].

Mammalian cells possess immune cells that express pattern recognition receptors named as Toll-like receptors (TLR). These receptors recognize foreign pathogen-associated structural patterns. This immune response can be activated either by TLR dependent or TLR independent pathways [79].

### 7.1. TLR Dependent Pathway

In TLR dependent pathway, thirteen TLR receptors have been recognized in humans as well as the mouse. Among these receptors, three TLR receptors, including TLR1, TLR2, and TLR3, have been identified to be triggered in response to siRNA [82]. TLR3 recognizes the duplex siRNA in a sequence-independent manner. Meanwhile, TLR7/8 recognizes the siRNA in a sequence-dependent manner. The example of sequence-dependent immune response by TLR7/8, the uridine, and guanosine-rich sequences with either UG dinucleotide or 5′-UGU-3′ potently trigger the innate immune response [82]. Avoidance of such sequences decreases the immune response [80]. Also, AU rich patterns have shown greater influence on TLR8 response [83]. Signaling by these intracellular TLR receptors requires acidification of endosomes along with maturation. Therefore, this endosomal acidification can be inhibited by the use of chloroquine or by buffering agents [84].

TLR3 possesses a specific gene structure among the TLR receptors and linked to its subfamily dissimilar from TLR7/8. At the cellular level, TLR3 expression in human leukocytes is vastly restricted to mature dendritic cells (mDCs). While in mice, TLR3 is found in a vast variety of immune cells. Recently, TLR3 expression on the cell surface has been noted in primary human endothelial cells such as lung, aorta, dermis, choroidal, and umbilical vein. TLR3 can also be present in several other cells, including epithelial, endothelial, and fibroblast lines [85]. Alexopoulou et al. demonstrated that dsRNA act as a TLR3 agonist by examining the response of splenocytes, macrophages, or bone marrow-derived DCs in TLR3 knockout mice to polyinosinic–polycytidylic acid (polyIC), a long synthetic dsRNA homolog [86]. TLR3 receptors on the surface of endothelial cells can also be activated by exogenous siRNA of 21 nucleotide base pairs in-vivo, resulting in the release of cytokines such as IL-12 and IFNγ. Unlike the various nucleic acid-sensing TLR receptors, that signal through the MyD88, TLR3 receptor signals through the TRIF adaptor protein [87]. TRIF signal is generated for the production of IFNβ along with IFNα4 by TANK-binding kinase-1 (TBK1), leading to the activation of transcription factor IRF3, which results in a positive feedback phenomenon to cause TLR3 signaling. This mechanism leads to the production of IFNβ and IFNα4 signals back to the cell, causing the up-regulation of IRF7 and other IFN-inducible genes. TLR3 activation via TRIF also activates RIP1 and TRAF6, leading to the activation of ATF, NF-κB, and c-Jun transcription factors along with the induction of inflammatory cytokines such as IL-6 and TNFα [88].

As discussed earlier, TLR7 and TLR8 recognize the siRNA in a sequence-dependent manner. It has been observed that plasmacytoid dendritic cells (pDCs), B cells, and monocytes express TLR7. At the same time, TLR8 is expressed by myeloid DC (mDC) and macrophages in humans [89]. TLR7 has been shown to detect single-stranded RNA (ssRNA), polyuridine, as well as synthetic ssRNA rich in uridine along with guanosine [90]. Similarly, TLR8 recognizes AU-rich and GU-rich ssRNA, and RNA viruses also trigger it. siRNA duplexes with GU-rich sequences or identified 5′-UGU-3′ motifs within particular siRNAs confer this highly immunostimulatory activity [81]. According to studies, it has been stated that TLR7/8 recognizes the siRNA in a sequence-specific manner [91]. Several immune-stimulatory signaling mechanisms are stated in Table 4.

Avoiding uridine and guanosine motifs may reduce a sequence-specific immune response. The replacement of siRNA motifs with adenosines may also abrogate the immune activation [97]. Interestingly, replacement of 2′-hydroxyl uridines with 2′-fluoro, 2′-O-methyl, or 2′-deoxy uridines blocks the immune activation. Thus, RNA immune recognition by TLRs can be prevented using 2′-ribose modifications of uridines [82]. The addition of 2′-O-Me uridine and guanosine into the sense strands of siRNA forms non-inflammatory siRNA without reducing their activity, and also protect the 5’ end of the guide strand that is vulnerable towards exonucleases [98]. As discussed earlier, chloroquine and bafilomycin A1 inhibit endosomal acidification and block the TLR3, TLR7, and TLR8 of siRNA without interfering with their RNAi activity [99].

### 7.2. TLR Independent Pathway

In TLR independent pathway, cytoplasmic RNA sensors such as retinoic acid-inducible gene 1 (RIG-1, also known as DDX58), melanoma differentiation-associated gene 5 (MDA-5), and dsRNA-binding protein kinase (PKR) are included [88]. It has been reported that RIG-1 binds to either ssRNA or dsRNA with uncapped 5’triphosphates, leading to the production of IFN. Therefore RIG-1 expression is readily upregulated in the para or autocrine manner, following the interferon response [100]. RIG-I and MDA5 activate the common downstream adaptor molecule MAVS. Activated MAVS then recruits multiple signaling molecules, including TRAFs, TBK1, and IRF3/7, leading to the transcriptional upregulation of type I interferons and other proinflammatory cytokines [101]. Finally, dsRNA-dependent PKR phosphorylates the serine along with threonine residues of the target protein. In humans, most of the cells express a low level of PKR and thus remain inactive. However, PKR binding to dsRNA leads to the formation of the dimer that ultimately undergoes autophosphorylation and subsequent activation of the pathway [102]. It has been suggested that the detection of dsRNA by PKR is a sequence-independent manner and interferon presence upregulates its expression [103].

## 8. Protein Binding

**Figure 4:**
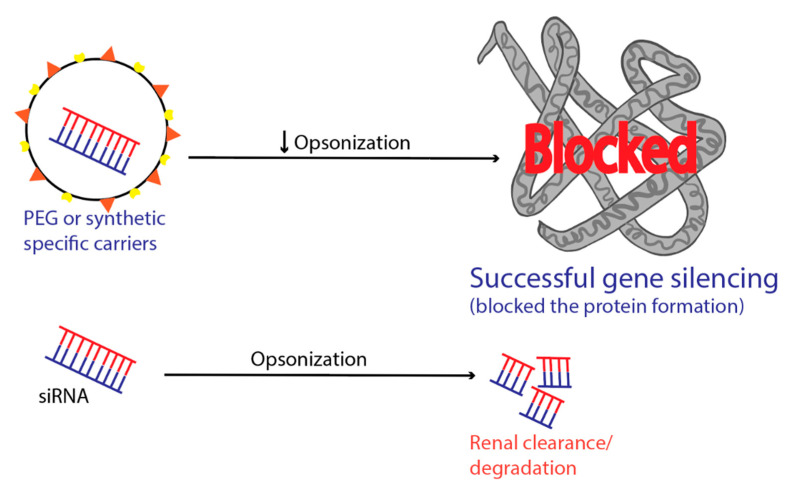
Surface modification of siRNA carriers to decrease opsonization, renal clearance, and degradation.

To achieve cell permeation of siRNA for effective concentration within the cell, a positively charged carrier is an essential requirement. Blood complement proteins and cell membrane proteins are usually negatively charged in the systemic circulation. The surface of the siRNA loaded carrier is positively charged, and it can be readily opsonized by opsonin proteins or may cause un-specific interaction with the cell membrane proteins [104]. Opsonins are essentially blood complement proteins or immunoglobulins. These blood complement proteins bind with the positively charged siRNA carriers through electrostatic interaction. The opsonization mechanism can occur anywhere in the blood circulation, completing from seconds to days. The opsonized carriers then undergo reticuloendothelial system (RES) filtration, and their cell surface binding leads to inflammation. The opsonization process makes siRNA carriers more susceptible to RES filtration and results in fast renal clearance (Figure 4) [55]. Opsonized carriers loaded RNA drugs accumulate in the liver and spleen and may cause toxic effects [105]. Thus, ultimately opsonization process reduces the therapeutic concentration needed for their efficient RNAi activity [55].

Surface modifications are the primary strategies to reduce the interaction between opsonin proteins adsorption and RNAi carriers. Coating of positively charge carriers with hydrophilic polymers such as PEG or specific synthetic polymers significantly decreases the opsonization along with unspecific binding [105,106]. For example, palmitate-avidin containing PLGA carrier adsorbed PEG-biotin on their surface, and the 10 kDa PEG-coated particle reduced protein adsorption by 75% [107].

## 9. RES Entrapment

As listed earlier, a major problem is the uptake of nucleic acid drugs by the RES. RES/mononuclear phagocytic system containing organs, including the liver, spleen, lung, and bone marrow, are saturated with the fenestrated capillaries along with phagocytic cells, macrophages, and monocytes [108]. It is thought that nanoparticles greater than 100 nm are trapped by RES in the liver, bone marrow, spleen, and lung, leading to degradation by activated monocytes and macrophages [108]. siRNA loaded nanoparticles undergoing the opsonization process are readily removed by the macrophages in RES [55]. Once the siRNA therapeutics reach the bloodstream, they have to be protected from the phagocytic cells of the mononuclear phagocyte system (MPS) [70]. The uptake of siRNA-nanoparticles from blood circulation to RES organs is quite a fast process. Still, these carriers are processed and eliminated slowly, resulting in prolonged retention of these particles in the filtering organs [55]. Somehow, the RES filtration process of specially siRNA-based therapeutics becomes helpful sometimes when the desired organ is a RES-rich organ [39].

Numerous factors, such as surface features, charge, and size of the carrier, may interfere with RES uptake and bio-distribution. It is thought that a surface with a negative charge is more susceptible to clearance from blood as compared to positive or neutral charged carriers. Surface modification is the primary strategy to bypass this barrier [39]. For example, siRNA carriers can be grafted with hydrophilic polymers such as PEG and other surfactant copolymers such as polyethylene oxide and poloxamers that give them stealth properties and cause them to remain in the blood for a prolonged time [109,110]. These modifications resist the protein adsorption, thereby protect the siRNA against opsonization and ensure cargo stability. These stealth properties are efficient for particles ranging from 70–200 nm. It is interesting to note that more pegylation may also neutralize the positive charge of the surface needed for the siRNA uptake into the cells [39]. Hence customized pegylation is required that causes the carrier to evade RES, but meantime does not compromise the cellular uptake of siRNA. For instance, if pegylation of siRNA-lipoplexes is increased from 1–2 to 5 mol% of PEG2000, it completely eradicates the siRNA-mediated gene silencing against PTEN protein in-vitro [111]. It is noteworthy that the pegylation strategy needs further exploration to identify the size and molecular weight of the PEG for designing an optimum siRNA delivery system.

## 10. Membrane Impermeability

**Figure 5:**
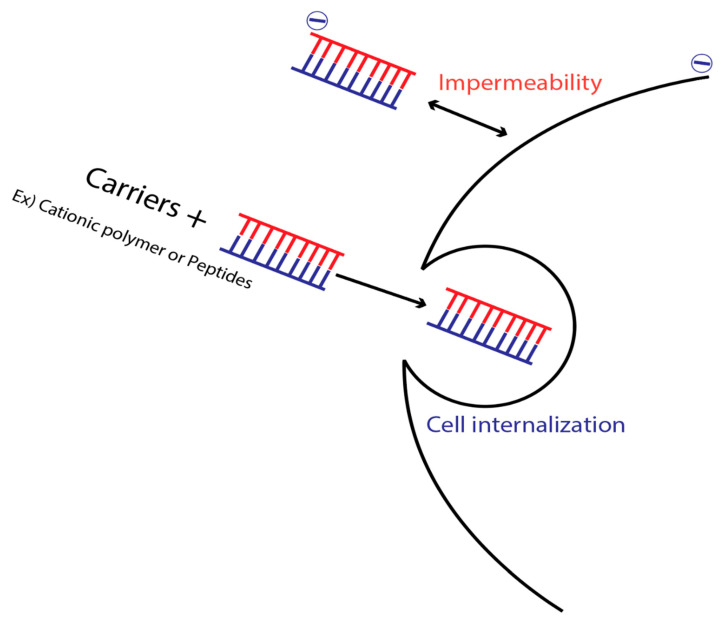
Carriers to overcome the impermeability of the siRNA.

As discussed earlier, naked siRNA cannot cross the plasma membrane because of its negative charge. Despite its small size, the negative charge and high hydrophilicity prevent siRNA from passing through the biological membrane. Hence, efficient delivery of siRNA needs modification to overcome this barrier [112]. In this context, carriers that enable efficient siRNA delivery are required. Complexation of siRNA with cationic polymers or lipids can mask the net negative charge of siRNA. Furthermore, these nanoparticles with positive charge interact with the negatively charged biological membrane, thus causing internalization [113]. Alternatively, efficient delivery of siRNA can be achieved by conjugating the siRNA with ligands, immunoglobulins, or aptamers that recognize specific antigens on target cells. When these conjugates bind to the cell, the siRNA uptake is done by receptor-mediated endocytosis resulting in endosomes formation with subsequent siRNA delivery into the cytoplasm [112]. Figure 5 illustrates this concept.

Due to the hydrophilicity and negative charge of the siRNA, various carriers are used, including cationic polymers and peptides. Peptides, in particular, have received specific attention because they show great promise as siRNA carriers, based on the diversity of their physiochemical properties and functions. Based on the function, several classes of peptides, including cell-penetrating peptides (CPPs), endosome-disrupting peptides, and non-covalent multifunctional peptide complexes, can be used [114]. CPPs, also referred to as protein transduction domains (PTD), can penetrate the plasma membrane of a cell and facilitate the delivery of various cargoes. Moreover, the cytotoxicity of CPPs is relatively low [115]. For instance, Tanaka et al. reported stearic acid conjugated a CPP demonstrated that injecting STR-CH_2_R_4_H_2_C peptide with siVEGF suppressed the tumor growth efficiently compared with naked siRNA or controlled group [116].

## 11. Endosomal Escape

Following the internalization of the siRNA, a major barrier remains to be its inability to escape endosomes. Thus, a carrier or modification that allows for the disruption of the endosomal membrane is essential for efficient endosomal escape and gene silencing by siRNA. Many recent delivery vehicles have taken advantage of the change from extracellular to the endosomal environment. Among the many differences, the change in pH is often utilized for efficient endosomal disruption and further release of siRNA once inside the target cell [117]. Protonable cationic polymers have been shown to increase delivery efficiency due to their high buffering capacity [45].

Several endosomolytic agents such as polymers, proteins, peptides, and small molecules like chloroquine can also be employed in siRNA carrier formulations to avoid the endosomal trap [118]. These carrier increases the counter-ion concentrations, leading to osmotic swelling, endosomal membrane rupture, and the subsequent escape into the cytosol [119]. A prime example of a positively charged polymer is polyethyleneimine (PEI), which can act as a “proton sponge” and facilitate the endosomal release of siRNA. The proton sponge effect facilitates the chloride ion influx and destroys the endosomes with subsequent release of contents into the cytoplasm [119]. The “proton sponge” effect or proton absorbance by buffering polymers such as PEI is known to increase the ATPase-mediated influx of protons and counter ions credited to their ability to prevent acidification [120]. As a result, the increased counter ion concentration inside the endosome leads to osmotic swelling, and ultimately endosomal membrane rupture, releasing the carrier and siRNA into the cytosol [121]. Although the PEI has this capability, one of the major drawbacks is its cytotoxicity, known to increase along with its physical size [121]. Another reported agent for causing effective endosomal escape is poly(lactic-co-glycolic acid) (PLGA) [122]. PLGA is a copolymer, biodegradable, biocompatible, and helps in RNAi activity of the siRNA. PLGA provides a sustained release to the siRNA with increased cellular uptake [122]. Especially, the layer-by-layer complex (nanoparticles) with PEI and PLGA increases the endosomal escape efficiency [122]. Another strategy for endosomal escape is protonation; two amino acids arginine and lysine get protonated in low pH that lead to destabilizing of cellular membranes. Lysine- and arginine-based polymers covalently linked to siRNA formulation have been used extensively to induce the release of siRNA from endosomes into the cytoplasm [123].

**Figure 6:**
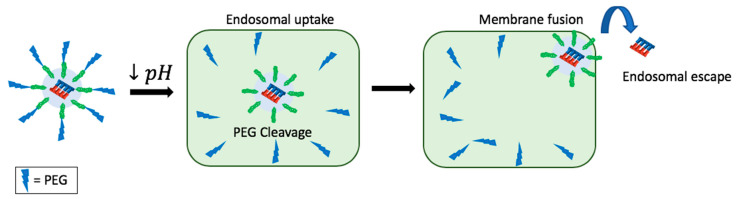
Mechanism of action of PEG-based cleavable polymeric system that responds to decreased pH in the endosomal environment.

Additionally, recent reports have cited the development of a PEG-based cleavable polymeric system, composed of nonlamellar highly fusogenic lipid nanoparticles that are further stabilized by surface-exposed PEG-conjugated vinyl ether lipids [124]. Its mechanism of action involves the hydrolysis of the vinyl ether bond at low pH inside of the endosomal environment, which results in the destabilization of the liposome and removal of PEG (Figure 6) [45]. This destabilization allows for the fusion between the liposome and endosomal membrane, ultimately releasing the siRNA in the cytosol efficiently, escaping the endosome [124].

Another strategy for promoting endosomal escape is the incorporation of fusogenic molecules that allows the carriers to fuse with the biological membrane following the release of endosomal contents into the cytoplasm [125]. For instance, a fusogenic lipid, 1,2-dioleoyl-sn-glycero-3-phosphoethanolamine (DOPE), has been extensively used in lipid-based siRNA carriers, that have a greater impact on endosomal escape. At low pH, DOPE undergoes a phase transition from the lamellar phase to the inverted hexagonal phase, leading to the destabilization of the endosomal membrane [126]. DOPE-PEG combination can also be used in the endosomal escape strategy [127].

**Figure 7:**
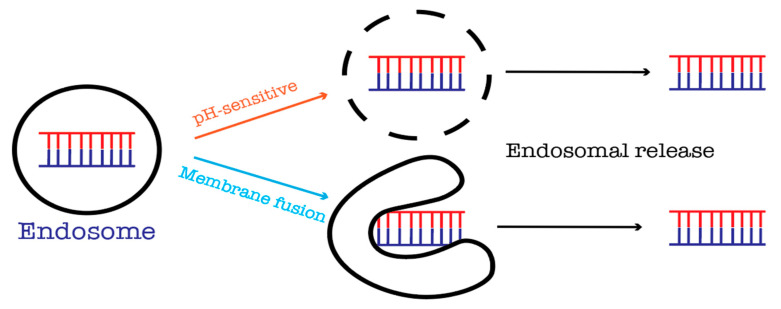
Ways to overcome endosomal entrapment.

Besides, fusogenic protein-based carriers have also been employed for rendering endosomal escape such as HA2 peptide, and a pH-sensitive fusogenic GALA peptide that changes their conformations in response to acidification of the endosome and triggers the endosomal release (Figure 7) [45].

## 12. Off-Target Effects

In terms of siRNA formulation to accumulate in tumors, specifically, it is only around 20 to 40% higher than that of normal tissue [128]. The enhanced permeation and retention (EPR) effect, which stems from leaky blood vessels in tumors, allows for the preferential uptake of the formulation in tumors compared to normal tissue but is not significant enough and may ultimately cause more harm than good in the long run [128]. Thus, to overcome these off-target effects, the use of surface-ligand modifications for siRNA formulations have recently been utilized for more targeted delivery of cargo. Many tumors and other diseases overproduce cell-surface receptors that can be preferentially targeted through the modification of a carrier with surface ligand modifications. To overcome the off-target effects, the receptor-targeted-delivery, conjugation with antibody, and chemical modification have been used.

First, receptor-targeted-delivery targets the specific receptors that are expressed in the specific type of tissue. This approach can directly deliver to the specific tissue and overcome the off-target effects. For example, researchers conjugated GalNAc ligand onto the siRNA to treat liver disease [129]. The drug based on GalNAc conjugation, givosiran, was approved by the FDA, as mentioned earlier in the introduction. GalNAc is one of the examples where modification was done at 3’ terminus of the sense strand. GalNAc conjugation bind to a receptor called ASGPR, also known as the asialogycoprotein receptor, which is responsible for uptake and clearance of circulation glycoprotein through a process of receptor binding to terminal galactose moieties followed by endocytosis [129]. This conjugate increased about 5-fold RNAi activity *in vivo*. During the experiment of targeting TTR mRNA, also known as transthyretin mRNA, the significant amounts of TTR mRNA were reduced in the liver cells, hepatocytes [130]. Besides, the PS (phosphorothioate) linkage modification of GalNAc conjugate improved the protection of 5’-exonuclease and required the lower dose of siRNA. Further research showed that not only triantennary but also the monovalent GalNAc conjugation improved the binding affinity and gene silencing efficiency [130]. The chemical modification may also prevent off-target effects [131]. Table 5 illustrates these chemical modifications. Particularly incorporating 2’-O-methyl modification of position 2 into one strand of the siRNA reduced the off-target effects and off-target mRNA [131]. As discussed earlier in Section 4, the use of transferrin protein to target the transferrin receptor, which is upregulated in various human tumor cells, is common [132]. Specifically, CALAA-01 made it onto the clinical stage and was tested in a cancer clinical trial [18]. Although the trials were discontinued after Stage I, fluorescence-based microscopy utilizing a CALAA-01 specific stain revealed the accumulation of the therapeutic within tumors but not in adjacent healthy tissue [18].

Additionally, in various tumors, it is possible to target the blood vessels supplying nutrients to the tumor through surface modification with peptide ligands. Among the many peptides, cyclic RGD (cRGD) is among the most commonly used modifications. cRGD can be isolated from in vivo tumor phage selection display and preferentially binds to *avβ3* and *avβ5* integrins. It is overexpressed on angiogenic endothelial cells of various tumor types and even on the tumors themselves [128]. cRGD is known to bind to integrin receptors 100-fold more tightly than linear RGD, making it the peptide of choice for surface modification [133]. A functionalized chitosan nanoparticle targeting PLXDC1 (a receptor highly expressed in tumor vasculature) inhibited ovarian tumor growth by about 90%. The modified cRGD nanoparticle showed to be 60% more effective than non-modified nanoparticles [134]. It is important to note that these peptide modifications to target blood vessel endothelial cells are yet to make it onto the clinical stage and is currently mainly being tested in murine models.

**Figure 8:**
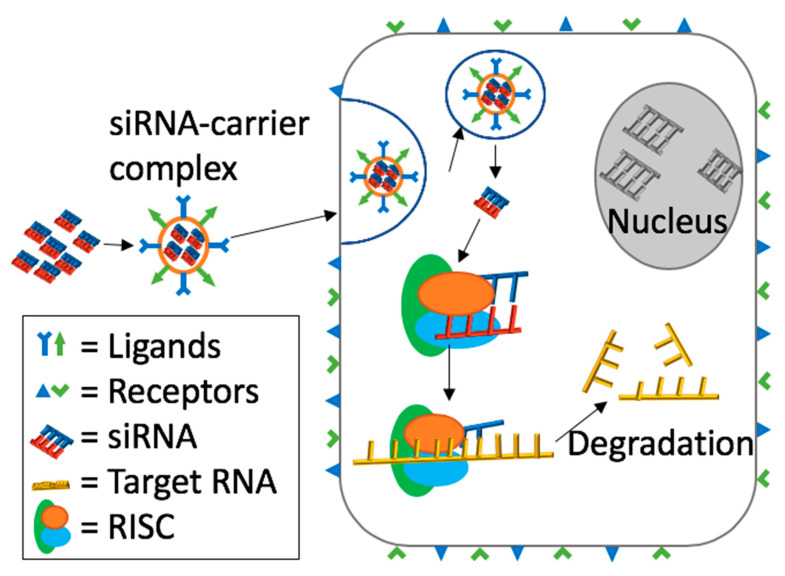
General mechanism of ligand-receptor mediated targeting and siRNA delivery. Nanoparticle carriers holding siRNA will be surface modified with complementary ligands of receptors overexpressed on the damaged or diseased cell. Once internalized, it will incorporate with the RNAi pathway and efficiently silence the gene of interest.

Finally, the antibody conjugation with siRNA may also minimize the off-target effects. During the experiment, antibody complex F105-p-siRNA was specifically delivered into HIV-infected Jurkat cells without triggering any interferon responses and delivering to adjacent cells [135]. Besides, another experiment of inhibiting the growth of B16 tumors, antibody-siRNA complex, was able to successfully prevent the growth, which explains that the antibody-siRNA complex has great therapeutic potentials [135]. Figure 8 explains general mechanism of ligand-receptor mediated targeting and siRNA delivery.

## 13. Other Strategies to Improve siRNA Effectiveness:

### 13.1. Aptamer-siRNA Conjugation

The aptamer is a single-stranded oligonucleotide with a three-dimensional structure that can be used to target cells. Their high affinity as well as specificity towards specific molecules, make it similar to monoclonal antibodies. However, aptamer shows several advantages over antibodies as they possess little-to-no toxicity and immunogenicity [136]. Both aptamer and the siRNA are nucleic acids, so both can be combined with simple covalent linkage or complementation (annealing). This combination of two has been mentioned as aptamer-siRNA conjugates [137]. Researchers can identify aptamers from libraries (>1014 shapes per library) by using a technique termed as SELEX (Systematic Evolution of Ligands by Exponential Enrichment) proposed by Gold and Szostak in 1990 [138]. Two different research groups first defined aptamer mediated siRNAs delivery. In both types of research, prostate-specific membrane antigen (PSMA) targeting RNA aptamers were used. PSMA is the first cancer cell-specific marker protein used for siRNA delivery with the help of aptamers. For critical metastatic cancer, an aptamer identifying alpha V and beta 3 (αVβ3) integrin were selected and linked to a siRNA against eukaryotic elongation factor 2, inhibiting proliferation and inducing apoptosis in target cells [139]. The last decade has seen several aptamers that bind to cell surface receptors, and upon ligand binding, it causes active internalization of cargoes, especially siRNA [140]. Table 6 illustrates the application of aptamers for delivery applications of nucleic acids.

### 13.2. Exosomes for siRNA Delivery

Another evolving innovative siRNA delivery vehicle is exosomes. Exosomes are naturally occurring RNA carriers that regulate the gene expression of recipient cells. Exosomes have been exploited for their use in delivering the siRNA within the cells [146]. They can bypass the several barriers encountered by other delivery vehicles for siRNA. Exosomes possess several advantages over other carriers, including greater delivery efficiency, membrane crossing capacity, biocompatibility, and non-immunogenicity [147,148]. Exosomes also show less toxicity and better safety while delivering the siRNA into the cells [149]. Besides, exosomes comprise of a specific protein and lipid composition that makes them suitable in delivering various cargoes directly to the cytosol through fusing with biological membranes [147]. Exosomes are nano-sized (40–100 nm) extracellular vesicles, and their design is based on endosome [150]. Once exosomes reach the desired target cells, their uptake can be mediated either endocytosis or fusion with biological membrane followed by internalization of siRNA in the cytosol [151]. Exosomes possess a slightly negative charge that is the reason for their stability in the blood. Besides, exosomes are more compatible with the immune system and do not trigger the immune response and have been reported to avoid off-target effecrs [152,153,154]. One of the most promising ability of exosome is to cross several biological barriers especially blood brain barrier (BBB), that warrant their potential use in several neurological disorders [155].

## 14. Innovations and Prospects

Besides its effectiveness, siRNA-based therapeutics face several challenges that limit their potential from bench to bedside. Nevertheless, numerous successful in-vitro studies and several investigations aimed at addressing the challenges for clinical application of siRNA therapeutics engenders great hope in this field. RNAi gains popularity in the scientific community quickly due to its potential to cause targeted knockdown of gene expression. Also, a great advancement in siRNA experiment protocols, easy availability of siRNA reagent kits, fast and efficient means to tailor siRNAs specific to desired sequences, latest bioinformatics software, and availability of the whole of the genome has made siRNA a lucrative tool for the cancer therapy as well as other intractable ailments [156]. A recent review delineates in detail the use of bioinformatics tools, software packages, and protocols in helping to design precise siRNA sequences for silencing desired genes and avoiding off-target effects [157]. Clinical evaluation of siRNA therapeutics began as early as 2004, but it was mostly for local administration. Now, the availability of FDA approved siRNA therapeutics bolstered hope in siRNA therapeutics [14]. Several approaches have been investigated to solve these challenges that have been thoroughly described in this review. For instance, chemical modification of the dsRNA has shown in numerous studies to attenuate immune response [158], permit resistance to endogenous endonucleases and exonucleases [159], improve sequence selectivity to reduce off-target RNAi activity [160], enhanced cellular permeability [161], and improved antisense strand selectivity by the RISC complex [162]. Another important factor in enhancing the effectiveness of siRNA therapy is the sequence selection. The antisense strand in dsRNA is the guide strand for the activation of RISC binding to the target mRNA. The sequences of the antisense strand is the single most important determinant of siRNA effectiveness. The sequence selection is not only important on-target potency but also it can profoundly minimize the off-target RNAi activity [163]. Avoiding the endosomal trap of siRNA and enhancing its escape for its cytoplasmic activity is a major challenge in RNAi based therapy [164]. Mostly, the siRNA, along with the carriers, enters the cells via endocytosis by interacting with anionic proteoglycans to form endocytic vesicles. After entry into the cell, the siRNA is entrapped in the endosomal vesicle, in case, the siRNA delivery system stays trapped in the vesicle, the late endosomal vesicle becomes acidified by membrane proton pump ATPase. It is relocated to the lysosome, where it is further acidified, resulting in degradation of the siRNA. Several carriers have been investigated to enhance the endosomal escape and increased transfection efficiency [45].

Keeping in view the progress made so far in addressing the barriers to siRNA therapy, numerous ongoing clinical trials in different stages, improved protocols for the systemic administration of siRNA treatment for targeted therapy, more mature clinical research and development, and the Good Manufacturing Practices, it is hoped that numerous siRNA based therapies will get approval from FDA in the years to come. Beyond these novel techniques and modifications in siRNA delivery systems for enabling the success of siRNA therapeutics, it is much likely that RNAi therapy has several paths for impactful innovations in challenging diseases over the next decade.

## 15. Conclusions

Most of the traditional drugs interfere with or modulate the disease-causing proteins. However, siRNA can silence the expression of the target proteins by interfering with the expression of target genes. Although it is almost two decades since the discovery of siRNA therapeutics, several challenges have limited the usefulness of siRNA therapeutics. With a lot of research in addressing those challenges, and recent emergence of siRNA drugs in the market has garnered a new hope in siRNA therapy. In this review, we have comprehensively described almost all the challenges pertaining to the success of siRNA therapy and the innovations and techniques that have been discovered to address those challenges. Many of the hurdles that were limiting the use of siRNA as an effective tool for the therapy of intractable disease has been overcome, several clinical trials and great interest by the Pharmaceutical as well as Biological Companies reveal that we will see some great breakthroughs in the therapy of cancer and other challenging diseases in the next decade.

## Figures and Tables

**Table 1:** Types of RNAi therapeutics.

Type of RNAi	Silent Feature of RNAi Therapeutics	References
miRNA	Stem-loop structure Binds imperfectly to mRNA and degrades many sets of similar target mRNA	[24]
shRNA	RNA with a tight hairpin turn Requires promoter to be expressed and must be located in the nucleus to act	[25]
siRNA	Short stretch dsRNA capable of degrading complementary mRNA Higher transfection efficiency and fewer obstacles.	[26]

**Table 2:** Barriers faced by siRNA to reaching site of Action.

Extracellular Barriers	Intracellular Barriers
Endogenous Nucleases	Endosomal trap
Elimination through kidneys due to small size	Arrival at the correct intracellular site of action (cytosol),
Impermeability to biological membranes	Off-target effects.
Entrapment by Reticuloendothelial System	
Plasma protein sequestration	
Capillary Endothelium Crossing	

**Table 3:** Characteristics of siRNA modifications.

Modification	Type of Modification	Outcome	Reference
Base	Internal uridine to rF (2,4-difluorotoluyl ribonucleoside) substitutions	Enhanced resistance towards serum nucleases	[57]
Sugar	2′-Deoxy-2′-fluoro-β-D-arabino, 2′-O-MOE modification, 2′-O-Me modification	Increase the half-life in the serum	[63]
	Locked nucleic acids	Longer half-life reduces immune activation	[64]
	4′-Thio modified the ring oxygen	Enhanced resistance towards nucleases	[65]
Backbone	Phosphorothioate (PS) modifications Morpholino oligomers	The longer half-life of duplex More potent	[66]

**Table 4:** TLR-dependent signaling in response to siRNA sequences.

Sequence	Signaling Mechanism	Type of Cytokines	References
5′-GUCCUUCAA-3′ motif	TLR8	Interferon-α	[92,93]
GU-rich sequence	TLR7	IFN-α	[94]
AU-rich sequence	TLR7 and TLR8	IFN-α, tumor necrosis factor (TNF)-α	[95]
5′-UGUGU-3′	TLR8	IFN-α	[92]
Repetition of uracil	TLR7	interleukin-6 and TNF-α	[96]

**Table 5:** GalNAc conjugation to overcome off-target effects.

GalNAc Conjugation	Modifications	Results	References
Triantennary	-Added PS linkages for the protection against the 5’-exonuclease.	-Reduced the off-target effects. -silenced mTTR in the liver cell (hepatocytes)	[131]
Monovalent	-2’ and 4’of the RNA’s ribose sugar backbone for GalNAc linker conjugation.	-Similar or better binding affinity and gene silencing efficiency compared to Triantennary GalNAc.	[130]

**Table 6:** Aptamers based on cell surface protein along with their delivery applications.

Receptor	Selection method	RNA/DNA	Delivery Applications	References
Transferrin receptor (TfR)	The extracellular purview of mouse TfR	RNA/DNA	Protein involved targeting lysosome	[141]
Nucleolin	N/A	DNA	Imaging variety of tumors	[142]
Tenascin C (TN-C)	Purified TN-C	RNA	Imaging variety of tumors	[139]
Prostate-specific membrane antigen (PSMA)	The extracellular purview of PSMA	RNA	Cellular imaging, siRNA delivery along with anticancer drug delivery	[143,144]
Epidermal growth factor receptor (EGFR)	The extracellular purview of EGFR	RNA	Delivery of nanoparticles	[145]
